# Sanitary Condition and Wants of Chicago

**Published:** 1862-07

**Authors:** N. S. Davis

**Affiliations:** Prof.


### CHICAGO MEDICAL EXAMINER.

N. S. DAVIS, M.D., Editor.

VOL. III. JULY, 1862. NO. 7.

### Original Contributions.

ARTICLE XIX.

### SANITARY CONDITION AND WANTS OF CHICAGO.

REPORTED TO THE CHICAGO MEDICAL SOCIETY, JUNE 6, 1862.

By N. S. DAVIS, M.D. & PROF.

In the April number of the Chicago Medical Examiner, I made some observations on the Metropolitan Health Bill, for the City of New York, then under discussion in the Legislature of that State.

What was then said in relation to New York, applies equally well to Chicago.

This is, not only, the great Commercial Metropolis of the North-west, inviting and receiving stated visits from merchants and traders in almost every town throughout this vast region; but its immense grain and lumber trade, brings it into almost equally extensive and direct contact with the agricultural population; while its Commercial Colleges, Theological Seminaries, Universities, and Medical Colleges, make it no less a resort for Students.

All these classes are directly interested in the sanitary condition and health of this city; first, individually, during their visits and stay here; and second, from their liability to receive and convey to their respective localities, whatever infectious or contagious diseases they may come in contact with while in the city. The naturally low and level condition of the city plot has given the locallity a traditionary character for unhealthiness, and an aspect, much of the year, calculated to strike the eye of strangers unfavorably. While the raising of the grade and the paving of the principal business streets has much improved their appearance; yet even this has left many vacant lots so much below the streets with a wet and dirty appearance that the impression of unhealthiness made upon strangers has scarcely been diminished. Let us go in whatever direction we may, whenever our city becomes the subject of conversation, unfavorable opinions in regard to its sanitary condition, founded on a simple passing observation of the circumstances to which we have alluded, are freely and confidently expressed. It matters not how erroneous these opinions may be in fact, they are efficient in detering strangers from choosing our city for residence, unless strongly impelled by purely business considerations.

It is thus evident, that strangers, non-resident business men holding frequent intercourse with us, students seeking education in our schools, and residents, are all directly interested in both the real and apparent sanitary condition of this city. We say *real* and *apparent* sanitary condition, because as we shall endeavor to show, there is a wide difference between the two; and yet both are worthy of careful consideration. There is no doubt but the location of Chicago, in its natural or unimproved state was unhealthy. When it was a mere trading post or military station known as Fort Dearborn, statistics show that there was an annual average number of attacks of malarious fevers, equal to 250 cases for evers 1000 inhabitants, and diarrhœa and dysentery prevailed in equal ratio.

It is well known that these endemic diseases continued to prevail annually, with but little amelioration until 1849. In the spring of that year, epidemic cholera made its appearance for the second time in this country. And the inhabitants of this city, then numbering but little more than 28,000, suffered as severely from that disease as almost any other, except those of New Orleans.

While this much dreaded scourge disappeared from nearly all the other cities of our country with the years 1849 and 1850, it continued to recur here, with considerable severity, during the summers of 1850, 1, 2, and 4. It is perfectly natural that all these facts, concurring with the natural appearance of the locality, should give it a very unfavorable sanitary reputation, both at home and abroad. Since 1854, an extensive system of sewers have been constructed, and the wholesome water of the Lake, which was previously supplied to only a small part of the city, has been carried to almost every part of it. The accumulation of vegetable matter on the surface of the prairie, surrounding the city on three sides, has been prevented by feeding and mowing, while the excess of water has been greatly diminished by ditching. These improvements, while effecting comparatively little change in the appearances which would strike a stranger or temporary visitor, have nevertheless produced a great change in the actual Sanitary condition of the city. The most reliable statistics accessible show that the annual ratio of mortality for the seven years ending with Dec. 31, 1854, was more than 1 in 30 of the population, while for the seven years intervening since that date, the average annual ratio of mortality has been less than 1 in 40. Thus making it, for the latter period, almost equal to that of the most healthy cities of the Union. It is true, that the common practice of measuring the sanitary condition of any community by the number of deaths in proportion to the population, is not entirely reliable. Because some diseases, as intermittent fever, catarrhal inflammations, and rheumatism, may be very prevalent annually and yet produce very few deaths; while other diseases, as pulmonary consumption, typhoid fever, and dysentery, would give a far greater number of deaths in proportion to the number of cases. But from close observation and constant practice in this city during the last thirteen years, I am sure that by both the tests just indicated, its sanitary condition will be found to have greatly improved during the last half of that time. Notwithstanding this, however, it is apparent to every intelligent physician, that still greater improvements are not only practicable, but imperatively demanded by the welfare of the citizens and the reputation of this as the Commercial Metropolis of the North-west. I will endeavor, as briefly as possible, to point out the more important of the improvements, and the measures which should be adopted for effecting them.

1st. Our system of sewerage should be rendered more efficient and valuable, by compelling the owners of vacant lots to so connect them with the street sewers as to cause a prompt removal of all the water falling on the surface of such lots; and by rigidly requiring all owners of houses to keep the rear of their lots, and especially the surface immediately under the houses, constantly free from standing water, by means of permanent connection with the street sewers. The splashing of water under the loose planks of the side-walks, the mud, and puddles of green water in the gutters, may impress the visitor or mere superficial observer, with a horrid idea of the salubrity of the city, but those are really almost harmless when compared with the surface water which is allowed to saturate the soil in the rear of, and under the dwellings; and in the latter place, often to stand in pools two-thirds of the year. It is from this source that the invisible aqueous vapor rises through the floors, dampening bedding, clothing, books, everything, indeed, in the house; and in the warm season, causing such things as are not in constant use, not only to become damp, but often also covered with a vegetable fungus, called "mould."

I am satisfied that much of the severe sickness which has occurred in this city, during former years, has originated directly from this source. Since the construction of permanent sewers, the evil has been greatly diminished; but a careful inspection will show that property owners are still, in many instances, extremely negligent in regard to this matter.

2d. Another improvement, closely allied to the foregoing, should consist in requiring the street commissioners to have all ditches, gutters, and entrances to cesspools and sewers carefully and thoroughly cleared of obstructions as soon as the frost is out of the way in the spring. The constant neglect of this, is really the cause of retaining much of the water, not only in the ditches, but on the surface of lots, and under side-walks and dwellings, until it is re-evaporated by the early summer heat. For years this matter has been almost entirely neglected throughout the city, except in the central business streets, until so late in the season that the oppressive mud of the ditches often mixed with the refuse of the kitchens, shoveled out upon the streets became at once exposed to a summer sun sufficiently hot to effect rapid decomposition, and fill the air with more offensive effluvia than though it had been left undisturbed. When ample facilities are given for the rapid escape of water, every shower through the spring and summer only tends to cleanse and purify the surface of the streets, alleys, and yards, while at the same time it flushes and washes out the sewers. But if the water is retained on the surface until it escapes by evaporation, it mascerates whatever decaying material exists, either on the surface or in the soil, and the rising vapor carries with it the products of decomposition to impregnate the air, and become an efficient cause of disease.

3d. In connection with sewers there is another item worthy of careful attention. It is the escape of offensive gases from the outlets and other openings of the sewers, in such quantity as to render the whole air of the vicinity extremely offensive to the smell, if not otherwise injurious. This is a matter particularly calculated to produce an unfavorable impression on strangers and recent residents. The turning of all these offensive gases into the river, has undoubtedly aided in producing its present impure and offensive condition. So far as the ordinary sewers contribute to this offensive condition of the river, I think the evil can be greatly mitigated, if not entirely removed, by a very simple process. It is well known that charcoal will absorb and render odorless, large quantities of the most offensive gases. Hence if all the main sewers were supplied, at proper distances, with grated ventilators, so constructed that a bushel of charcoal could be placed in each ventilator, the gases generated in the sewers, instead of being forced on to their exit in the river, would escape as fast as generated, through the charcoal in the ventilators and be thereby rendered both odorless and harmless.

The charcoal in the grates could be readily changed as often as experience should prove it to be necessary. This arrangement, with the attention to clearing of the street gutters, ditches, cesspools already described, and a little more attention to the frequent flushing of sewers by fresh water from the fire hydrants, would effectually and permanently secure the river and the city from injury by our system of sewerage.

I am, by no means, disposed to attribute the present black and extremely offensive condition of the water in the river, altogether, to impurities derived from the street sewers. First, because there is nothing in the nature or composition of the ordinary sewerage matter that could impart the peculiar black color at present seen in the river water. Second, because neither the extent nor connections of the street sewers have been materially altered for the last *two* years; and yet the present peculiar condition of the river was not manifested until the last four or five months. Third, because there are other sources of the most foul impurities that require a very rigid investigation.

The most important of these sources are: the slaughter-houses, the hog-yards, and the distilleries located on the two branches of the river; and the waste water saturated with sulphurous gases from the gas-works. From direct inquiries, I am satisfied that most if not all those engaged in slaughtering and meat-packing, on the river branches, intend to have both the blood and offal carried entirely away from the river, but it is to be feared that those who are entrusted to do the work, carelessly allow much to enter the river which should have been excluded. The city cannot exercise too vigilant a supervision of these establishments; and certainly no hog or cattle yards connected with distilleries should be allowed in such positions as to turn their offensive refuse into the water.

If all these controllable sources of filth and putrefaction had been guarded against by an efficient system of supervision—if the street gutters, ditches, and cesspools had been kept thoroughly free—if the sewers had been properly ventilated with the aid of charcoal; and if regular attention had been given to stated flushing of the sewers by fresh water from the hydrants, the river would not at this time present the horribly offensive condition in which it really is. Yet it is highly probable that other and additional means for maintaining the purity and healthfulness of the river must now be resorted to. In devising such additional means, the two all-important objects are, efficiency and permanence. To secure the first, it is necessary that a current should be established and maintained, sufficient to clear the channel of the river from all foul deposits. Four methods of doing this have been suggested.

First, it has been proposed to construct a dam across the branches of the river, with a suitable gateway; and when a sufficient *head* of water has been raised, open the gateway and let the water above suddenly sweep out the channel.

There are two fatal objections to this proposition. In the first place, there is no point on either branch of the river where a dam could be made to raise the water to a sufficient height for the purpose, without overflowing the banks and the prairie, so as to create a greater evil than the one for which it is to constitute a remedy.

In the second place, the small amount of water flowing into either branch during July, August, and September, (the very time when flushing is most needed,) would render it wholly unreliable as a source of supply at that season of the year.

The second proposition is to open a communication between the North Branch and the Desplains River, by which enough of the water of the Desplains should be turned into the North Branch, to maintain an efficient current towards its mouth.

This proposition involves two inquiries:

1st. Is the supply of water through the Desplains at such a level, and always in such *quantity*, as to insure the needed current in our river?

2d. Would the diversion of the water, sufficient for our purpose, from the Desplains interfere with the just rights and privileges of the inhabitants occupying the district of country through which that river runs?

We have not the result of actual surveys before us, in regard to the relative height of the Desplains and the North Branch of the Chicago River, but assuming that the former is sufficiently elevated, is its supply of water abundant at all seasons of the year? We fear not. For in the dryest part of every summer and autumn, the water in its channel becomes so low that if the whole was turned into the North Branch its presence would hardly be felt; and yet that is the very season when we need the most efficient supply for the purpose of purifying our river.

If at such a season of the year, all the water of the Desplains is turned from its natural channel, it would doubtless prove a serious loss and inconvenience to all the inhabitants living near it, below the point of diversion.

Without any certainty of obtaining a sufficient amount of pure water at the season of the year when most needed, and the strong probability of meeting opposition and perhaps expensive litigation, we think the proposition to interfere with the natural course of the water in the Desplains scarcely worthy of serious consideration.

The *third* remedy which has been proposed for relieving the river of its offensiveness, is, to open a channel sufficiently deep for the current of water to be permanently changed from the South Branch of the Chicago River, southward to the Illinois River; thereby converting our river into an outlet instead of an inlet to the Lake.

The proposition which is being urged upon Congress, to construct a Ship Canal, connecting the Lake at this point with the Illinois River, has been looked to with hope for the practical accomplishment of this plan or remedy. The construction of a Ship Canal, however, is a work requiring several years for its completion, and the expenditure of many millions of dollars; and is altogether too remote and uncertain to be relied upon as a sanitary measure for this city.

The *fourth* plan for keeping the river free from offensiveness, is that proposed as a part of the original system of sewerage. It consists in opening a channel from the Lake to the South Branch of the river, as far south as practicable, through which an abundant supply of water, to flush and purify the river, can be at all times furnished from the Lake, by means of a stationary engine and elevating water-wheel.

This plan has the advantage of being certainly effectual in its results; of being always fully under the control of the proper authorities of the city; and of not involving an expenditure of money greater than the city could bear.

These advantages are certainly sufficient to give it the preference over all other projects, as a permanent measure for preserving a proper sanitary condition of the Chicago River.

If the measures already alluded to in this report, for keeping the street gutters, ditches, lot and house drains always clean and free from obstructions; the main sewers properly ventilated and flushed at suitable intervals of time from the street hydrants; the cattle and hog yards connected with distilleries removed from the banks of the river branches; *the immense amount of sulphurous gases turned from the gas-works into the Monroe Street sewer, stopped;** and the sand-bar at the mouth of the river dredged out, so as to permit a more free channel, not only for commerce, but for the egress and ingress of currents of water, there would be very little need of any other special means for preserving a proper sanitary condition of our river, at least for some years to come. But if such special or extra means should be found necessary, then a water-wheel, like the one at Bridgeport for feeding the canal, and an engine sufficient to move it, with a channel from the Lake to the South Branch of the river, would constitute the most efficient, permanent, and economical plan that could be devised for that purpose.

4th. Next in importance to an improvement in the condition of the river, is the furnishing of a purer quality of water throughout the city for ordinary use. The water of Lake Michigan, when properly obtained, is as pure and wholesome as is to be found in any part of the world. By the present arrangement of the Water Works, however, the water is taken from the Lake so near the shore and so far within the densely populated part of the city, that it not only becomes riley with every storm, but it often becomes perceptibly impregnated with

* We have not any doubt but the greater part of the offensive smell, and the peculiar black color of the river water during the present season, is derived from the refuse of the Gas-works, which was last autumn turned into the Monroe Street sewer.

the offensive effluvia escaping from the mouth of the river. This should be immediately remedied, either by extending the supply-pipe much farther into the Lake, or along the shore to some point north of the old cemetery, the neighborhood of which should be thoroughly guarded by efficient legislative enactment, against the present or future establishment of distilleries, breweries, slaughter-houses, cattle-pens, or any kind of manufacturing business that could possibly affect the purity of the water.

5th. Another improvement of great importance bearing on the sanitary condition of the city, would be the rigid enforcement of the ordinance requiring in all cases a certificate showing the name, age, residence, and cause of death, before a body could be *interred* in any of the cemeteries connected with the city. The system or practice at present pursued by the City Sexton, is perfectly valueless for all purposes, except merely the gross number of deaths. If the ordinance requiring certificates to be procured was rigidly enforced, and the certificates immediately returned to some one, not only competent to judge whether they were properly made, but also capable of tabulating them correctly at proper intervals, we could soon ascertain both the absolute and relative healthiness of every street, ward, or district in the city. Statistics thus prepared would not only be valuable to sanitary science generally, but would render invaluable aid in ferreting out, and removing from among us, the active causes of disease and death. Of course, the statistics thus obtained, together with a full report on the sanitary condition of the city, and the means for improving it, should be made to the Mayor and Council, at least once a-year.

I know of no city, except *Chicago*, with a population of more than 110,000, that has neither a health officer, board of health, or any other official sanitary organization. Not long since, one of our daily morning papers contained a whole column of editorial, founded on the supposition that there was no provision for medical attention to the sick poor of the city, and calling on the Mayor to appoint a "City Physician" for that purpose. That editorial was altogether erroneous.

The poor of the city, whether sick or well, belong to the County of Cook, and are exclusively under the care of the Board of Supervisors. That Board has for several years past, appointed two physicians; one to reside at or near the county poor-house, and attend the inmates of that institution, and another, resident of this city, whose duty it is specially to attend such sick poor within the city as are entitled to aid from the county.

These are certainly medical officers enough to take care of the *sick poor*, and consequently we want no "City Physician" on their account.

What the city does need, however, for the purpose of ensuring efficient attention to the important matters discussed in this report, is a thoroughly competent Medical Health Officer, whose duty it should be to keep up a constant inspection and supervision of everything relating to the sanitary condition and mortality of the city. He should be required to report to the Mayor and Council promptly, all matters requiring special or immediate attention, and make a general report, suitable for publication, once a-year. To him all certificates of death should be returned, that the statistics furnished thereby might be included in his annual report. He should be selected solely with reference to his thorough qualifications as a Sanitary Superintendent and adviser, without the slightest reference to his political opinions or party attachments. Such an officer would be of inestimable value to the city.

